# Exothermic Effects and Viscosity of Oxide Melts Formed During High-Temperature Reduction of Cr–Mn Ore Systems

**DOI:** 10.3390/molecules31030575

**Published:** 2026-02-06

**Authors:** Yerbolat Makhambetov, Sultan Kabylkanov, Saule Abdulina, Armat Zhakan, Azamat Burumbayev, Zhadiger Sadyk, Amankeldy Akhmetov, Zhalgas Saulebek, Ruslan Toleukadyr, Arnat Smagulov, Onuralp Yücel

**Affiliations:** 1 Chemical-Metallurgical Institute Named After Zh. Abishev, Karaganda 100030, Kazakhstan; armat.medetuly@gmail.com (A.Z.); a.burumbayev@tttu.edu.kz (A.B.); sadzhad03@gmail.com (Z.S.); aman1aotero@gmail.com (A.A.); zhalga1998@gmail.com (Z.S.); rus.toleukadyr@gmail.com (R.T.); arnatsmagulov@gmail.com (A.S.); 2 International School of Engineering, East Kazakhstan Technical University Named After D. Serikbayev, Ust-Kamenogorsk 070000, Kazakhstan; sabdulina@edu.ektu.kz; 3 Department of Metallurgical and Materials Engineering, Istanbul Technical University, Istanbul 34469, Turkey; yucel@itu.edu.tr

**Keywords:** multicomponent oxide melts, Cr–Mn system, exothermic reduction reactions, slag viscosity, high-temperature, Arrhenius analysis, FactSage

## Abstract

This study investigates the exothermic effects and viscosity properties of multicomponent oxide melts during the high-temperature reduction of low-grade Cr–Mn ores. Unlike previous thermodynamic-focused research, this work provides experimental evidence of transient exothermic responses and correlates them with melt properties. High-temperature experiments identified pronounced exothermic effects in the 800–1600 °C range. Phase analysis (XRD, SEM–EDS) confirmed effective Cr and Mn reduction into Fe–Cr–Mn–Si alloys with minimal residual oxides in the slag. Effective viscosity, measured via the electrovibrational method at 1400–1650 °C, decreased monotonically with temperature. Arrhenius analysis was applied to determine activation energies and crystallization onset temperatures (T_cr_). The results indicate low viscosity and high thermal stability of the slags, ensuring efficient metal–slag separation. These findings confirm the technological feasibility of using low-grade ores for Fe–Cr–Mn alloy production and provide a basis for optimizing industrial smelting.

## 1. Introduction

The development vector of 21st-century metallurgy has shifted fundamentally toward the production of high-performance materials characterized by minimized production costs and stringent environmental compliance [1,2,3,4,5,6]. Current market dynamics dictate new paradigms: the rising costs of alloying components and the implementation of carbon taxes compel steel producers to seek alternative technological routes for cost reduction without compromising the functional quality of the final product.

One of the most pressing challenges is the steady decline in the grade of mined chromium and manganese ores. The depletion of high-grade deposits necessitates the direct smelting of low-grade, multicomponent raw materials without costly pre-enrichment stages. However, the utilization of such lean ores significantly alters the physicochemical properties of the resulting slags—most notably their viscosity. An increase in impurity levels often leads to erratic viscous behavior, which hinders efficient metal–slag separation and results in substantial losses of valuable elements to the waste phase.

**Figure 1:**
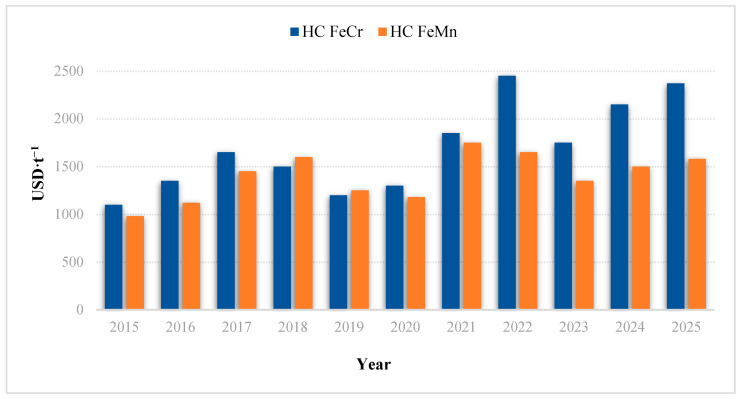
Global price dynamics of conventional high-carbon FeCr and FeMn (2015–2025).

**Figure 2:**
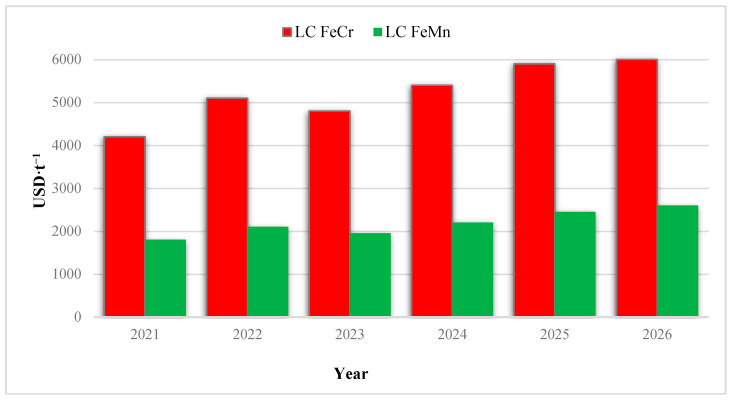
Global price dynamics of conventional low-carbon FeCr and FeMn (2021–2026).

The situation is further exacerbated by the unprecedented price volatility of key ferroalloys. By the end of 2025 (Figure 1), the cost of high-carbon ferrochrome (FeCr) surpassed 2100 USD/t, while ferromanganese (FeMn) stabilized at approximately 1550 USD/t. Projections for 2026–2027 suggest that prices for low-carbon (LC) grades will reach new historic highs due to the global energy crisis and the full implementation of the European Union’s Carbon Border Adjustment Mechanism (CBAM) (Figure 2).

Starting in January 2026, the export of ferroalloys with a high carbon footprint will be subject to additional taxation [7]. This regulatory shift renders traditional, energy-intensive separate production of FeCr and FeMn less competitive compared to the integrated production of complex Cr–Mn master alloys.

Multicomponent oxide melts represent complex high-temperature systems whose macroscopic properties are fundamentally governed by the structural organization of the oxide network [8,9,10,11]. The phase evolution and viscosity characteristics of such melts directly reflect alterations in the degree of polymerization of structural units [12,13,14,15,16,17]. In systems based on the CaO–SiO_2_–MgO–Al_2_O_3_ quaternary, viscosity is regarded as the most structure-sensitive parameter [18,19,20].

Supporting studies emphasize that as the composition of oxide systems becomes increasingly complex, thermal effects exhibit non-stationary behavior, necessitating a rigorous thermodynamic analysis of reaction energetics [21,22,23,24]. According to established literature, the viscosity of oxide melts shows extreme sensitivity to temperature fluctuations and chemical variations [25,26,27,28]. Shifts in the ratios of major oxide components lead to pronounced changes in both viscosity and crystallization temperature, reinforcing the critical role of viscous properties in the phase evolution of these multicomponent systems [29,30,31].

Despite extensive research, the thermal effects of reduction reactions have largely been studied in isolation from the macroscopic properties of the resulting melts. Previously, the authors demonstrated the thermodynamic feasibility of producing complex chromium–manganese master alloys via the aluminosilicothermic method [32]. This approach proposes the use of complex reducers such as AlSiMn (aluminum–silicon–manganese) and FeSiAl (ferrosilicoaluminum), synthesized from locally sourced high-ash coals [32]. The utilization of these alternative reducers instead of traditional, expensive analogs significantly reduces the production cost of the master alloy, making it economically superior to the separate production of LC FeCr and LC FeMn.

However, in previous studies, the implementation of this process was justified solely through thermodynamic modeling. Experimental validation of the impact of intense exothermic effects—accompanying aluminosilicothermic reduction—on the viscosity and structural evolution of multicomponent oxide systems has remained unexplored until now. Without a fundamental understanding of how localized thermal “micro-explosions” during reduction affect slag fluidity, it is impossible to ensure efficient phase separation and high metal recovery under industrial conditions.

The present study aims to bridge this gap. We transition from theoretical calculations to the experimental investigation of the thermal behavior and viscosity of Cr–Mn system melts. This paper examines the dynamics of viscosity changes directly during the heating process and the progression of reduction reactions. Such an integrated approach not only validates thermodynamic predictions but also establishes the actual kinetic parameters required to minimize metal losses and optimize the thermal balance of the smelting process. This work paves the way for the development of import-substituting, energy-efficient technologies for Fe–Cr–Mn master alloy production, adapted to current market challenges and environmental standards.

## 2. Results and Discussion

### 2.1. Analysis of the Reduction Reaction Energetics and Thermal Effects

In all three experimental variants, high-temperature reduction was successfully initiated, leading to the synthesis of a Cr–Mn-containing metallic phase. The process was characterized by distinct gravitational separation between the metallic and oxide phases, confirming that the system achieved a stable metal–slag equilibrium under the applied conditions.

**Figure 3:**
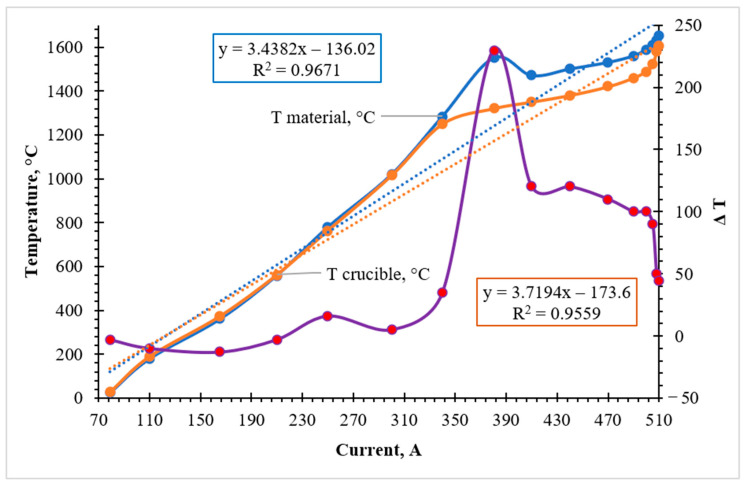
Temperature–current profiles illustrating the exothermic thermal response during the aluminosilicothermic reduction of chromium-bearing ore (Variant 1).

**Figure 4:**
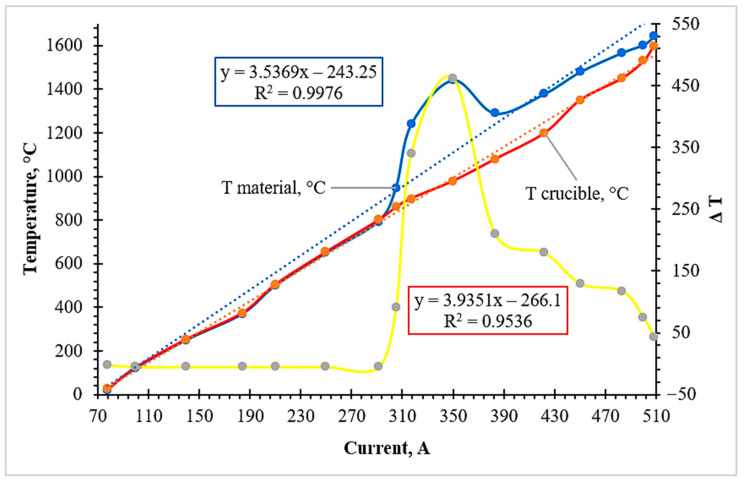
Temperature–current profiles illustrating the exothermic thermal response during the complex reduction of chromium and iron–manganese ore mixture using FeSiAl reductant (Variant 2).

**Figure 5:**
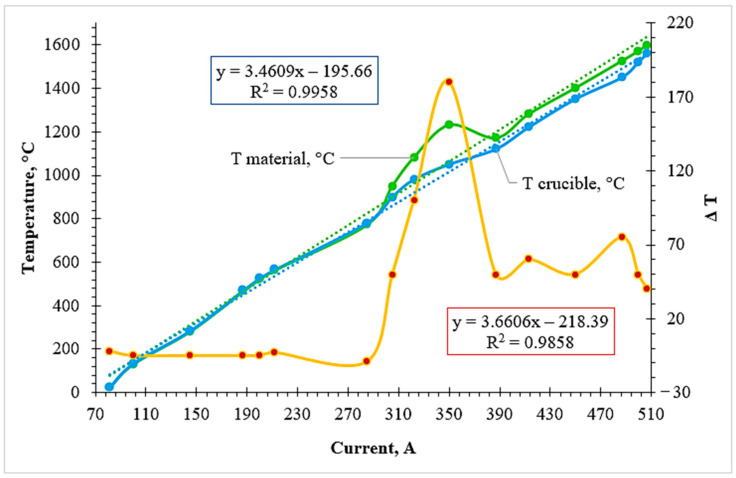
Temperature–current profiles and thermal behavior during the reduction of Kerege-Tas iron–manganese ore using FeSiCr dust (Variant 3).

To evaluate the energetics of the reduction process and identify characteristic exothermic responses, the temperature–current profiles (Figure 3, Figure 4 and Figure 5) were subjected to detailed thermal analysis. Unlike standard heating curves, these dependencies reflect the non-stationary nature of the chemical interactions occurring within the melt as a function of the inductor current (A). The analysis of these profiles allows for the identification of specific “ignition” points where internal chemical energy begins to dominate the external induction heating, providing a mechanistic understanding of the thermal evolution of the system. 

The temperature–current profiles obtained for the first experimental variant (Cr-ore + AlSiMn reductant + lime) provide critical insight into the energetic evolution of the system (Figure 3). At the initial heating stage (up to ~1250 °C), the thermal state was governed strictly by the external power input, with the temperatures of the melt (T_material_) and the crucible (T_crucible_) increasing linearly as the inductor current was raised from 80 to 300 A. This coordinated heating indicates the absence of significant internal chemical heat generation during the pre-reduction phase.

A pronounced shift in the thermal regime was observed in the current range of 340–380 A. Within a 12–14 min interval, while the inductor current was held in a quasi-stationary regime, a rapid temperature surge of the melt from 1285 to 1550 °C was recorded. This sharp increase of 250–270 °C cannot be attributed to the induction heating alone, providing direct experimental evidence of an internal exothermic heat source.

From a mechanistic perspective, this thermal pulse is driven by the high negative enthalpy of the metallothermic reduction of chromium oxides by the active components of the aluminosilicate reductant (Al and Si). Since manganese is introduced into the system in its metallic form, the exothermic effect is primarily localized within the chromium reduction reactions:

1. Cr_2_O_3_ + 2Al = 2Cr + Al_2_O_3_ (ΔH_298_ = −548 kj/mol);
2. 2Cr_2_O_3_ + 3Si = 4Cr + 3SiO_2_ (ΔH_298_ = −365 kj/mol).

The sharp increase in the temperature difference (ΔT) between the melt and the crucible at ~380 A confirms that the heat release occurred directly within the reaction zone. Following the peak, the temperature stabilized at ~1470 °C as the chemical energy was dissipated and absorbed by the surrounding slag phase. At the final stage, the heating was again controlled by the induction power as the current reached 510 A, with the final melt temperature reaching approximately 1650 °C.

Thus, the exothermic effect in Variant 1 manifests as a short-lived transient thermal pulse. This localized energy release is functionally significant: it provides the necessary heat to facilitate the rapid depolymerization of the oxide network, thereby lowering the viscosity of the multicomponent melt and ensuring efficient gravitational separation of the synthesized Cr–Mn metallic phase from the slag.

The temperature–current profiles recorded for the second experimental variant (chromium ore + iron–manganese ore + FeSiAl + lime) provide a comprehensive view of the thermal behavior of the system during complex reduction (Figure 4). During the initial heating phase (up to 900 °C), the system followed a stable induction heating regime where the melt (T_material_) and crucible (T_crucible_) temperatures increased linearly with the inductor current.

A transition in the kinetic regime was observed starting from ~950 °C. Initially, a slight plateau in the heating rate occurred, which is attributed to the endothermic energy requirements for initial slag formation and the rearrangement of the oxide lattice. However, as the current reached the 300–350 A range, a powerful exothermic pulse was triggered. The melt temperature exhibited a sharp jump from ~1240 °C to ~1440 °C (a ΔT of 200 °C) while the external power input remained quasi-stationary. This provides definitive experimental evidence of internal chemical heat generation.

Unlike Variant 1, where the reduction processes were primarily limited to chromium and iron oxides, the introduction of iron–manganese ore in this variant significantly alters the energetic profile. The presence of manganese oxides expands the range of exothermic interactions. From a mechanistic standpoint, this thermal surge is the cumulative result of the following reactions:

Aluminothermic stage (Primary heat pulse):

1. 3MnO + 2Al = 3Mn + Al_2_O_3_ (ΔH_298_ = −519 kj/mol).
2. Cr_2_O_3_ + 2Al = 2Cr + Al_2_O_3_ (ΔH_298_ = −548 kj/mol).

Silicothermic stage (Thermal sustainment and slag formation):

1. 2MnO + Si = 2Mn + SiO_2_ (ΔH_298_ = −185 kj/mol).
2. 2Cr_2_O_3_ + 3Si = 4Cr + 3SiO_2_ (ΔH_298_ = −365 kj/mol).

The participation of silicon (Si) in the reduction of manganese and chromium oxides ensures sustained heat release following the initial aluminum-driven pulse. The reaction products, specifically SiO_2_, are instrumental in forming a fluid silicate-based slag. This combined energy release promotes the rapid depolymerization of the oxide melt, significantly reducing its viscosity. This reduction in viscosity is the key factor that allows the freshly reduced metallic droplets to coalesce and settle through the slag, ensuring high recovery rates of the Cr–Mn–Fe alloy.

After the reaction front passed, the temperature stabilized at ~1290 °C before the induction heating brought the final melt to ~1640 °C at 508 A. Thus, the thermal behavior in Variant 2 is characterized by a transient, high-intensity thermal pulse that optimizes the conditions for phase separation and alloy formation.

The temperature–current profiles for the third experimental variant (ferromanganese ore + FeSiCr dust + lime) reveal a distinct thermal regime under a constant frequency of 50 Hz (Figure 5). Up to a current of approximately 290 A, the heating followed a stable linear trend, governed predominantly by the external induction energy input.

In the 290–350 A range, a moderate transient thermal pulse was recorded, causing the melt temperature to rise from ~1080 °C to a peak of ~1230 °C. Compared to the previous variants, this exothermic response is significantly less intense and exhibits a shorter duration.

Mechanistic Analysis: The lower intensity of the exothermic effect in Variant 3 is fundamentally linked to the physical and chemical state of the charge components. Unlike Variants 1 and 2, where chromium was introduced in the oxide form (requiring highly exothermic reduction), Variant 3 utilizes FeSiCr dust. In this technogenic material, chromium is already present in its metallic state. Consequently, it does not participate in exothermic redox reactions; instead, the metallic chromium acts as a thermal “sink,” consuming a portion of the generated heat for its own melting and superheating.

Therefore, the observed thermal pulse is attributed exclusively to the silicothermic reduction of manganese oxides from the Kerege-Tas ore by the silicon present in the reductant:

1. 2MnO + Si = 2Mn + SiO_2_ (ΔH_298_ = −185 kj/mol).

Despite the moderate nature of this heat release, the thermal energy is sufficient to initiate the depolymerization of the multicomponent oxide melt, thereby reducing its viscosity. This provides the necessary kinetic conditions for the coalescence of Cr–Mn metallic droplets and their subsequent gravitational separation from the slag. Following the reaction stage, the system stabilized, reaching a final equilibrium temperature of 1600 °C at 510 A.

In summary, the thermal and kinetic analysis of the three experimental variants was focused primarily on the reduction of chromium (Cr) and manganese (Mn) oxides. While secondary reactions occur within the multicomponent system, these primary transitions are the fundamental drivers of the system’s energetics and the formation of the metallic phase. The experimental data confirms that the localized thermal pulses, regardless of their intensity, are sufficient to trigger the depolymerization of the slag and ensure high recovery rates of the target elements.

To verify the efficiency of the reduction process, the final metallic and slag phases were subjected to rigorous chemical analysis. The elemental compositions were determined using the wet chemical method in accordance with GOST standards (for low-carbon FeCr and FeMn alloys). This analytical approach ensured high precision in measuring the distribution of Cr and Mn between the alloy and the oxide melt. The results of the chemical analysis for all three variants are presented in Table 1.

Analysis of the chemical composition of the metallic phase shows that the obtained Cr–Mn-containing samples represent multicomponent alloys of the Fe–Cr–Mn system with a high total content of the main transition elements. For all investigated variants, a stable redistribution of chromium and manganese into the metallic phase is observed, which is reflected in the close and reproducible composition of the obtained products.

The obtained data indicate the formation of a metallic matrix enriched with transition metals, while local compositional variations point to non-equilibrium crystallization conditions caused by the intense progression of reduction reactions and subsequent cooling of the melt. The contents of Cr and Mn compounds in the slag phase remain limited, as confirmed by the low residual concentrations of the corresponding oxides in the smelting products.

### 2.2. XRD and SEM Analysis

**Figure 6:**
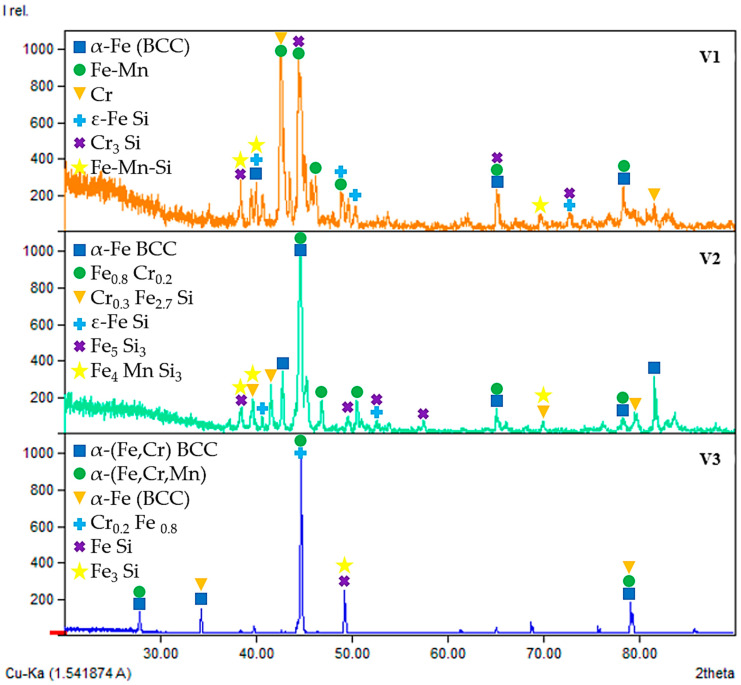
X-ray diffraction patterns and phase identification of the V1, V2, and V3 samples.

**Figure 7:**
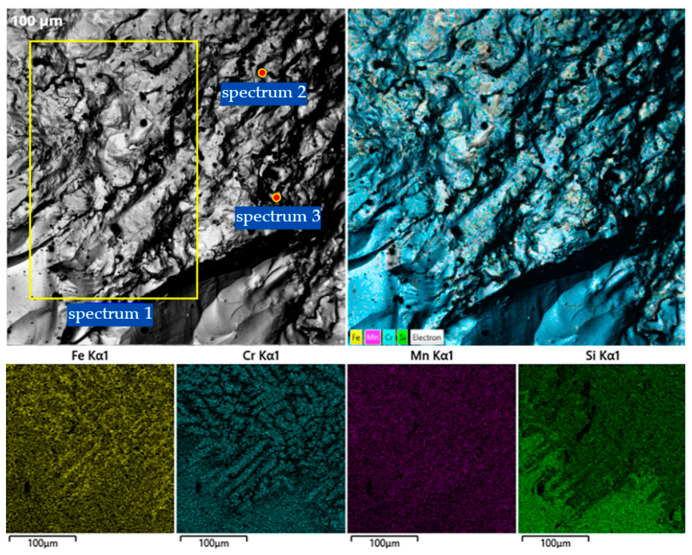
SEM images and EDS elemental maps (Fe, Cr, Mn, and Si) of the Cr–Mn metallic phase obtained using the AlSiMn reductant.

**Figure 8:**
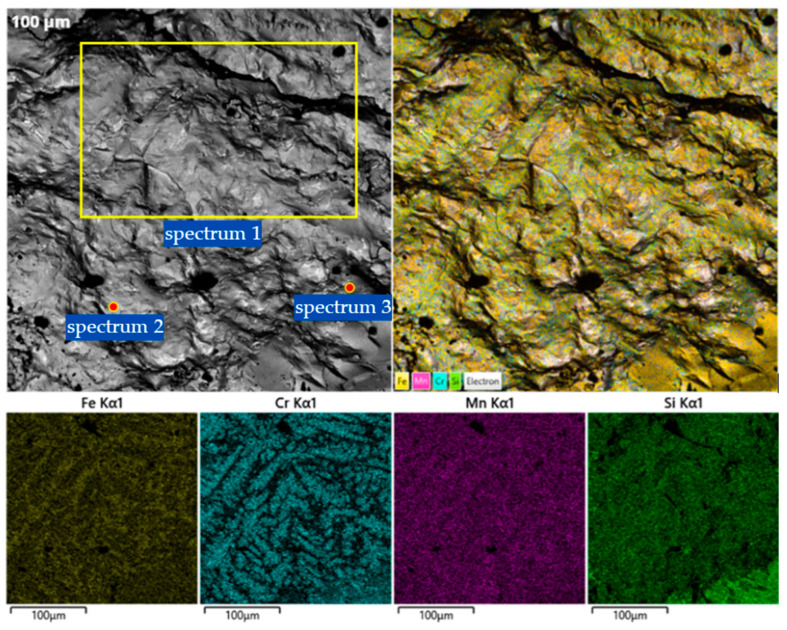
SEM images and EDS elemental maps (Fe, Cr, Mn, and Si) of the Cr–Mn metallic phase obtained using the FeSiAl reductant.

**Figure 9:**
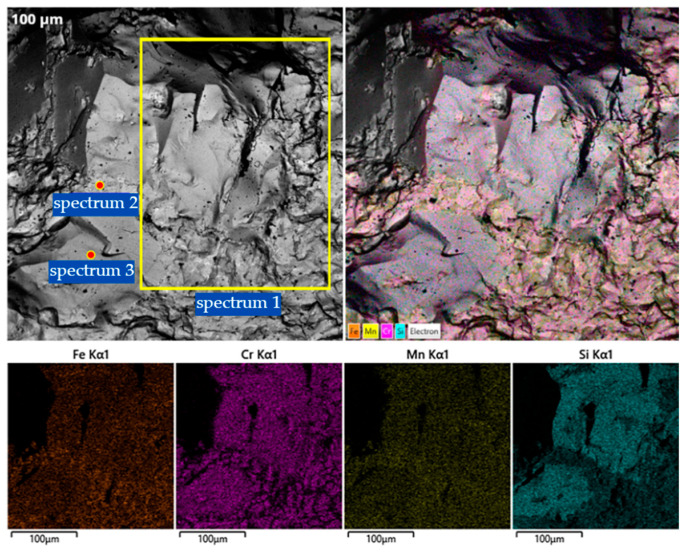
SEM images and EDS elemental maps (Fe, Cr, Mn, and Si) of the Cr–Mn metallic phase obtained using FeSiCr dust as the reductant.

To determine the phase composition and microstructural features of the obtained Cr–Mn-containing metallic phase, a combination of X-ray diffraction and qualitative scanning electron microscopy methods was employed. The combined use of XRD and SEM makes it possible to identify crystalline phases, assess their morphological distribution characteristics, and reveal features of non-equilibrium crystallization caused by the intensive progression of reduction reactions and specific thermal conditions. The obtained data (Figure 6, Figure 7, Figure 8 and Figure 9) provide the basis for interpreting the chemical composition of the metallic phase and for further analysis of the relationships between phase state, microstructure, and the conditions of high-temperature experiments.

#### 2.2.1. XRD Analysis of the Metallic Phase

**V1.** The XRD pattern of the metallic product obtained in Variant V1 indicates a clearly multiphase structure. The dominant diffraction peaks correspond to α-Fe with a body-centered cubic (BCC) lattice, as evidenced by intense reflections in the 2θ range of approximately 42–45°, as well as higher-angle reflections near 65° and 78–80°. These peaks are partially broadened and split due to the formation of Fe–Mn solid solutions, indicating the incorporation of manganese into the iron lattice.

In addition to the Fe-based phases, distinct reflections of metallic chromium are observed, confirming the effective reduction of chromium oxides. The presence of ε-FeSi, Cr_3_Si, and Fe–Mn–Si silicide phases is also evident from characteristic peaks in the 38–41° and 45–50° ranges. The formation of these silicides demonstrates the active participation of silicon in the reduction process and reflects non-equilibrium crystallization under conditions of rapid heat release. Overall, the phase assemblage of Variant V1 corresponds to a Cr–Mn–Fe alloy with a significant fraction of secondary silicide phases.

**V2.** The metallic phase obtained in Variant V2 is characterized by a different phase balance. The XRD pattern is dominated by α-Fe-based solid solutions alloyed with chromium, as indicated by intense BCC reflections and their systematic shifts relative to pure iron. The strongest peak at approximately 44.7° (2θ) confirms the prevalence of the Fe–Cr metallic matrix.

Compared to Variant V1, Variant V2 exhibits a more pronounced development of iron–chromium and iron–chromium–silicon silicide phases, including Cr_0.3_Fe_2.7_Si, ε-FeSi, and Fe_5_Si_3_ (xifengite). These phases are identified by multiple reflections in the 38–42°, 50–55°, and 70–80° regions. The occurrence of Fe–Mn–Si silicides and trace manganese-containing phases indicates partial participation of manganese in silicide formation rather than extensive incorporation into the Fe lattice. This phase composition suggests a stronger influence of silicon-driven reactions and a higher degree of chemical heterogeneity in the metallic product.

**V3.** In contrast to Variants V1 and V2, the XRD pattern of Variant V3 reveals a comparatively simpler phase composition. The metallic product is predominantly composed of α-Fe-based solid solutions with minor alloying by chromium and manganese, as reflected by sharp and well-defined BCC diffraction peaks. The reduced number and intensity of secondary reflections indicate a lower fraction of silicide phases.

Only weak reflections corresponding to FeSi and Fe_3_Si are detected, suggesting limited interaction between silicon and the metallic melt under the thermal conditions of Variant V3. The absence of pronounced Cr_3_Si or complex Fe–Mn–Si silicides implies a more equilibrium-like crystallization behavior and a lower degree of chemical segregation during solidification.

**Comparative discussion.** A comparison of the three variants demonstrates that the phase composition of the metallic products is strongly governed by the reduction conditions and the availability of active reductants. Variant V1 exhibits the most complex multiphase structure, combining Fe–Mn solid solutions, metallic chromium, and several silicide phases formed under highly non-equilibrium conditions. Variant V2 is characterized by a Fe–Cr-dominated metallic matrix with an increased contribution of iron–chromium–silicon silicides, reflecting the enhanced role of silicon in the reduction process. Variant V3 shows the simplest phase assemblage, dominated by Fe-based solid solutions with minimal silicide formation.

#### 2.2.2. SEM Analysis of the Metallic Phase

To complement the XRD findings, SEM combined with energy-dispersive X-ray spectroscopy (EDS) was performed on the metallic product of V1 (Figure 7). The microstructure analysis reveals a highly heterogeneous, multiphase morphology, which directly correlates with the complex diffraction pattern observed in the XRD analysis.

Microstructural features the SEM micrographs show a well-developed crystalline structure characterized by dendritic formations and intermetallic inclusions. This confirms the non-equilibrium crystallization process mentioned earlier, where rapid thermal shifts lead to the segregation of various phases. The elemental mapping displays a non-uniform distribution of Fe, Cr, Mn, and Si, indicating the formation of distinct chemical zones within the alloy.

Elemental composition (EDS Results) the quantitative EDS analysis (Table 2) of selected local areas (Spectra 1–3) provides specific insights into the phase distribution:

- Spectrum 1 (Cr-rich matrix): This region is dominated by chromium (69.63 wt.%) with significant iron (19.21 wt.%) and manganese (6.88 wt.%). This composition supports the XRD detection of metallic Cr reflections and the formation of α-(Fe,Cr) solid solutions.
- Spectrum 2 (High Mn-Si zone): A sharp increase in manganese (21.32 wt.%) and silicon (6.73 wt.%) is observed here. This local chemistry corresponds to the Fe–Mn–Si and Cr_3_Si silicide phases identified in the 38–41° and 45–50° (2theta) ranges of the XRD pattern.
- Spectrum 3 (Fe-Cr-Mn balance): This area shows a more balanced distribution of iron (29.11 wt.%) and chromium (44.39 wt.%) with high manganese (22.51 wt.%), confirming the presence of the complex Fe–Mn and BCC α-Fe based solid solutions.

The microstructural evolution and elemental distribution of V2 (Figure 8) exhibit distinct differences from V1, directly supporting the phase shifts observed in the XRD patterns. The transition from a multi-component Cr-rich system to a more dominated iron–chromium matrix is evident in both the morphology and the localized chemical analysis.

Microstructural Observations SEM imaging of Variant V2 (Figure 8) reveals a heterogeneous surface with clearer grain boundaries and specific clusters of silicide inclusions. The elemental maps for Fe, Cr, Mn, and Si show that while iron and chromium form the primary matrix, silicon and manganese are concentrated in specific secondary phases. This microstructural heterogeneity explains the multiple reflections of complex silicides identified in the XRD analysis.

Quantitative EDS Interpretation The point EDS analysis for Variant V2 (Table 3) clarifies the chemical nature of the phases:

- Spectrum 1 (Fe-Cr-Mn matrix): This area shows a high concentration of iron (48.36 wt.%) and chromium (23.38 wt.%), which aligns with the XRD results showing the prevalence of α-(Fe,Cr) BCC and Fe_0.8_Cr_0.2_ solid solutions.
- Spectrum 2 (Mn-rich phase): A significant concentration of manganese (38.22 wt.%) and silicon (6.11 wt.%) is detected. This chemical signature correlates with the Fe_4_MnSi_3_ and manganese-containing silicide phases noted in the diffractogram.
- Spectrum 3 (High Chromium area): This region is dominated by chromium (52.16 wt.%) and iron (36.37 wt.%), supporting the identification of chromium-rich silicides like Cr_0.3_Fe_2.7_Si and Cr_0.2_Fe_0.8_ observed in the higher angle XRD reflections.

The microstructure of V3 (Figure 9) is noticeably more uniform compared to the previous samples, which is consistent with the simplified phase composition shown in the XRD patterns.

Microstructural observations the SEM images of Variant V3 exhibit a more homogeneous metallic matrix with a significant reduction in the size and quantity of secondary silicide inclusions. The elemental maps show a more even distribution of iron and chromium, indicating a closer approach to equilibrium crystallization conditions compared to Variants V1 and V2.

Quantitative EDS interpretation point EDS analysis of V3 (Table 4) reflects a stabilized chemical composition across the metallic product:

- Spectrum 1 (Main Matrix): Characterized by high manganese (39.29 wt.%) and iron (39.98 wt.%) content with moderate chromium (16.72 wt.%), confirming the formation of complex α-(Fe,Cr,Mn) solid solutions identified by XRD.
- Spectrum 2 (Mn-enriched zone): Shows a substantial manganese concentration (61.32 wt.%), which aligns with the intense reflections of manganese-incorporated phases in the diffraction study.
- Spectrum 3 (Balanced alloy zone): Demonstrates a balanced distribution of iron (41.77 wt.%) and manganese (27.03 wt.%) with a low silicon content (4.88 wt.%), correlating with the minor FeSi and Fe_3_Si reflections detected in the 48–50° (2theta) range.

**Summary of Phase and Microstructural Evolution.** The comparative analysis of the three variants demonstrates a clear correlation between the crystalline phases identified by XRD and the microstructural features observed via SEM/EDS. In V1, the complex multiphase diffraction pattern, including distinct Cr_3_Si and ε-FeSi reflections, is fully validated by the high degree of chemical segregation and the presence of Cr-rich dendritic zones in the SEM micrographs. V2 represents an intermediate state where the matrix shifts toward an iron–chromium base, with EDS confirming that manganese and silicon are primarily concentrated in specific silicide clusters like Fe_4_MnSi_3_ and Fe_5_Si_3_, as detected in the corresponding XRD patterns. Finally, Variant V3 exhibits the most homogeneous structure, where XRD and EDS maps reflect a stable α-(Fe,Cr,Mn) solid solution with minimal secondary inclusions.

Ultimately, these results confirm that the phase composition and morphology of the Cr–Mn metallic products are highly sensitive to the specific reduction conditions defined for each variant. The presence of silicon (ranging from 2.16 wt.% to 6.73 wt.% across all variants) acts as a key driver for silicide formation. While the AlSiMn reductant ensured high efficiency in chromium and manganese recovery, the variations in thermal regimes and reactant ratios between V1, V2, and V3 directly influenced the final phase distribution. The transition from the complex, non-equilibrium silicide-rich structure of V1 to the more stabilized solid solutions of V3 demonstrates that the degree of alloying and structural homogeneity can be precisely controlled. This consistency across XRD and SEM/EDS methods provides a reliable basis for interpreting the relationship between experimental parameters and the resulting alloy properties.

### 2.3. Viscosity of the Slag Melts

To elucidate the influence of thermal effects and phase composition on the behavior of the oxide phase at high temperatures, the viscosity properties of slags formed during reduction reactions in the Cr–Mn system were investigated. Slag viscosity is considered an integral parameter sensitive to chemical composition, oxide network structure, and temperature regime, allowing relationships between phase transformations, the energetics of reduction processes, and macroscopic melt behavior to be established.

In the present study, the viscosity behavior of the slag phase was determined using a combination of experimental high-temperature measurements and thermodynamic modeling. This integrated approach provides a comprehensive assessment of the effects of slag composition and exothermic thermal effects on viscosity and enables comparison of experimentally observed system behavior with calculated trends derived from equilibrium models.

The viscosity of the investigated slags was calculated using the FactSage 8.4 software package, which allows evaluation of the physicochemical properties of multicomponent oxide melts based on thermodynamic models and phase equilibria. Experimental viscosity values were obtained using an electrovibrational viscometer installed in a resistance furnace with a graphite heater, enabling assessment of the actual slag fluidity under conditions close to metallurgical practice.

**Figure 10:**
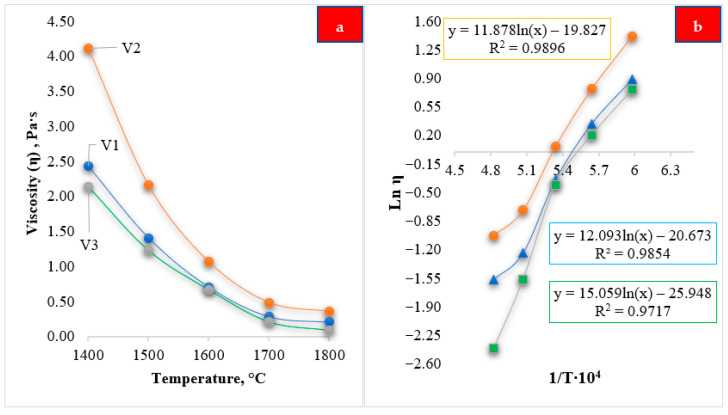
Temperature dependence of viscosity for slags of different compositions (V1–V3): (**a**) variation in viscosity (η) as a function of temperature; (**b**) linearization of the temperature dependence in ln(η) − 1/T coordinates.

At the first stage, the temperature dependence of slag viscosity was analyzed based on data obtained using the FactSage software (Figure 10a,b). For the three slag compositions considered, characteristic trends in viscosity variation were identified in the temperature range of 1400–1800 °C. The calculated results show that the viscosity of all investigated slags decreases monotonically with increasing temperature, which is attributed to gradual thermal disruption of the oxide structural network, a reduction in the degree of polymerization, and increased mobility of liquid-phase components.

As shown in Figure 10a, the viscosity of all investigated slags decreases monotonically with increasing temperature, which is a characteristic feature of oxide melts. This behavior is associated with the gradual thermal disruption and depolymerization of the oxide structural network, leading to increased mobility of structural units and ions in the liquid phase. Among the studied compositions, slag V2 exhibits the highest viscosity values over the entire investigated temperature range, whereas slags V1 and V3 show lower viscosities, indicating differences in their chemical composition and structural organization.

Figure 10b presents the linearization of the experimental and calculated data in ln(η)–1/T coordinates, demonstrating an approximately linear relationship with high correlation coefficients (R^2^ > 0.97). This confirms that the temperature dependence of slag viscosity within the investigated temperature range is adequately described by an Arrhenius-type equation. Differences in the slopes of the fitted lines indicate variations in the apparent activation energy of viscous flow, which are governed by the degree of polymerization of the oxide network and the ratio of network-forming to network-modifying oxides.

However, the FactSage software calculates viscosity only for an idealized homogeneous liquid phase and does not account for the possible presence of solid inclusions, microcrystalline phases, or structural heterogeneity of real melts. Therefore, to correctly describe the viscous state of slags under metallurgical conditions, the effective viscosity determined experimentally was used in this study.

For this purpose, slag samples were subjected to high-temperature measurements using an electrovibrational viscometer installed in a resistance furnace with a graphite heater. Pre-crushed slag samples (30–50 g) were placed in a molybdenum crucible (inner diameter 18 mm, outer diameter 30 mm, height 65 mm) positioned in the constant-temperature zone of the furnace.

After complete melting of the sample in the temperature range of 1550–1650 °C, the melt was stirred with a molybdenum rod for approximately 5 min to ensure chemical and thermal homogeneity. Subsequently, a molybdenum probe (spindle) with a diameter of 2 mm and a length of 40 mm was introduced into the central part of the crucible using a screw lifting mechanism to a depth of 10–12 mm from the melt surface.

**Figure 11:**
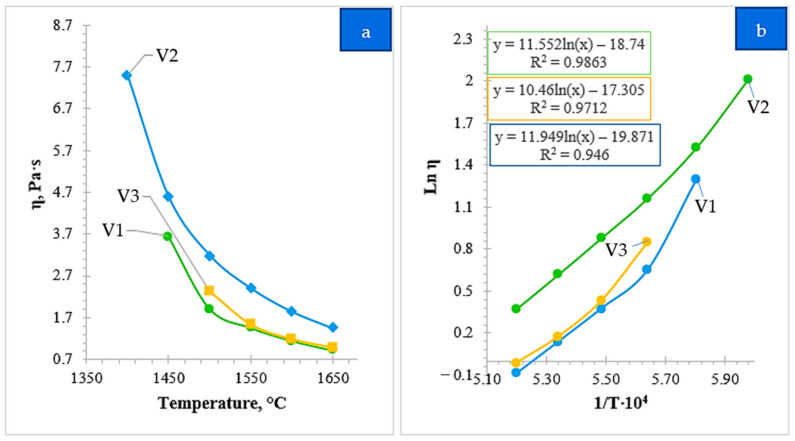
Temperature dependence of the effective viscosity of slags of different compositions (V1–V3): (**a**) variation in effective viscosity (η) as a function of temperature; (**b**) Arrhenius representation of the viscosity data in ln(η) − 1/T coordinates.

Viscosity measurements were carried out under homogeneous liquid conditions during controlled cooling at a rate of 3–5 °C·min^−1^ until the onset of solidification. Although the measurements were continuous, specific data points were selected for Figure 11 to facilitate a clear comparison between the experimental results for different compositions (V1–V3) and the linearized plots.

The temperature in the furnace working zone was monitored using a tungsten–rhenium thermocouple protected by a corundum sheath, with the measuring junction positioned in close proximity to the crucible bottom, ensuring high accuracy of melt temperature determination.

Comparison of experimentally determined effective viscosity with FactSage-calculated values revealed discrepancies caused by the influence of melt structural heterogeneity and the possible presence of solid phases. The obtained results confirm that the use of effective viscosity provides a more adequate description of slag behavior under conditions close to real metallurgical processes and enables reliable assessment of slag fluidity and technological suitability.

Figure 11a shows the temperature dependence of the effective viscosity for the three investigated slag variants (V1–V3), determined experimentally using the electrovibrational method. The temperature ranges for measurements (ranging from 1400 °C to 1650 °C) were individually selected for each variant based on their specific crystallization onset temperatures. For all systems, a monotonic decrease in viscosity with increasing temperature is observed, which is attributed to the thermally induced restructuring of the melt. At elevated temperatures, the breakdown of bridging bonds in the silicate–aluminate network occurs, reducing the degree of polymerization and increasing the mobility of modifying cations (Ca^2+^, Mg^2+^, Fe^2+^, Mn^2+^, etc.).

In the temperature range of 1400–1450 °C, the differences between the slag variants are most pronounced. Variant 2 (Curve 2) exhibits the highest effective viscosity values (up to ~7.5 Pa·s), indicating a more highly polymerized structural network with a larger fraction of complex silicate chains. Variants 1 and 3 show lower viscosity values in this range, suggesting a more open structural network. As the temperature rises above 1550 °C, the viscosity curves of all three variants converge, indicating a transition to a near-homogeneous state where the influence of structural heterogeneity and microcrystalline inclusions is minimized.

Figure 11b presents the experimental data in Arrhenius coordinates (ln_η_ − 1/T). While high linearity is observed in the high-temperature regions (R^2^ = 0.94–0.98), the plots exhibit a noticeable deviation from strict linearity as the temperature approaches the solidification point. This “curvature” in the Arrhenius plots is explained by the transition from a purely viscous flow to a heterogeneous state, where the onset of crystallization increases the apparent activation energy of the flow. This behavior is characteristic of real metallurgical slags and reflects the systematic increase in energy barriers for ion transport during cooling.

The variation in the slopes of the Arrhenius plots confirms that the temperature sensitivity of viscosity is governed by the slag chemical composition, particularly basicity and the ratio of network-forming to network-modifying oxides (CaO, MgO). Thus, the combined analysis of Figure 11a,b demonstrates that the viscosity behavior of the investigated melts is controlled by the degree of polymerization and the strength of ionic bonds within the oxide network under conditions closely mimicking metallurgical practice.

Table 5 summarizes the Arrhenius equation parameters, the activation energy of viscous flow (E_a_), and the crystallization onset temperatures (T_cr_) for the three investigated slag variants. These parameters were determined based on experimental effective viscosity data processed in lnη–1/T coordinates, as well as the analysis of viscosity anomalies identified on the temperature dependences.

The crystallization onset temperatures (T_cr_) were determined from characteristic inflection points on the effective viscosity versus temperature curves. At these points, the formation of the first solid phases in the melt begins, structural homogeneity is disrupted, and a sharp increase in the rate of viscosity growth is observed. According to the obtained results, T_cr_ values for variants 1, 2, and 3 are 1415, 1380, and 1435 °C, respectively.

The Arrhenius equations were obtained by fitting the experimental effective viscosity data and provide a quantitative description of the temperature dependence of the viscosity properties of the investigated melts. The coefficient B = E_a_/R characterizes the energy barrier associated with ion transport and structural rearrangement of the slag matrix. The calculated activation energies of viscous flow are 189.8 kJ·mol^−1^ for variant 1, 175.8 kJ·mol^−1^ for variant 2, and 161.7 kJ·mol^−1^ for variant 3.

The gradual decrease in activation energy from variant 1 to variant 3 reflects weakening of the melt structural network and a reduction in the degree of polymerization. Variant 1 is characterized by the most robust silicate–aluminate structure, whereas variant 3 exhibits a more mobile and thermally sensitive structural organization. Variant 2 occupies an intermediate position between these limiting cases.

The combined analysis of experimentally determined effective viscosity, Arrhenius parameters, and crystallization onset temperatures demonstrates that all three slag compositions exhibit low viscosity, high thermal stability, and a wide temperature range of homogeneous liquid-phase existence in the working temperature interval of approximately 1600 °C. Despite differences in E_a_ and T_cr_, all investigated variants provide favorable conditions for efficient metal–slag separation and stable smelting operation, confirming their technological suitability for producing Cr–Mn ligatures.

**Figure 12:**
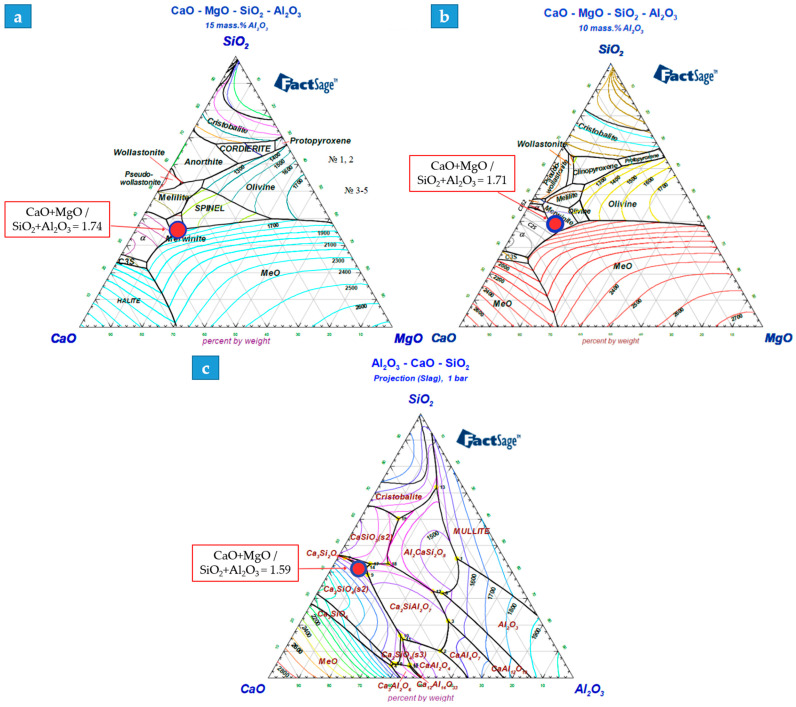
Phase diagrams of the investigated slag systems: (**a**) Variant 1; (**b**) Variant 2; (**c**) Variant 3. The markers indicate the positions of the experimental compositions relative to the liquidus surface and eutectic regions.

To interpret the experimental viscosity data, the investigated slag compositions were projected onto the phase diagram (Figure 12a–c). The analysis shows that Variant 1 (V1) is located at the boundary of the Merwinite/Periclase fields, with a liquidus temperature in the range of 1550–1600 °C. This positioning within a relatively fluid mineralogical zone explains its stable viscosity at operating temperatures.

Variant 2 (V2) is situated in a transition zone beyond the Merwinite field, with a higher liquidus temperature of 1600–1650 °C. The shift toward this more refractory region correlates with the observed increase in effective viscosity and a higher activation energy (E_a_), as the melt structure becomes more prone to polymerization and early phase precipitation during cooling.

Variant 3 (V3) lies at the boundary of the Rankinite/Belite fields (liquidus ~1600 °C). Despite the presence of these phases, the composition maintains high fluidity, which is consistent with the experimental results showing the lowest crystallization onset temperature (T_cr_). This phase-based evidence confirms that all three variants are optimized for smelting, with V1 and V3 providing the most favorable conditions for efficient metal–slag separation.

## 3. Materials and Methods

### 3.1. Raw Materials

In this study, low-grade ores from Kazakhstan were used as the primary sources for transition metal compounds. Specifically, chromium ore from the Kempirsay deposit and ferro-manganese ore from the Kerege-Tas deposit were utilized to form multicomponent oxide systems. These raw materials represent a strategic resource for the production of complex alloys due to their specific mineralogical compositions.

To promote chemically active reduction environment, low-cost silicon–aluminum-based reductants derived from high-ash coal processing were employed. These included complex alloys such as FeSiAl and AlSiMn, as well as FeSiCr dust, which is a finely dispersed byproduct generated during the crushing stage of ferro-silico-chromium production. The use of these secondary materials and inexpensive reductants is aimed at improving the economic efficiency of the alloying process.

**Figure 13:**
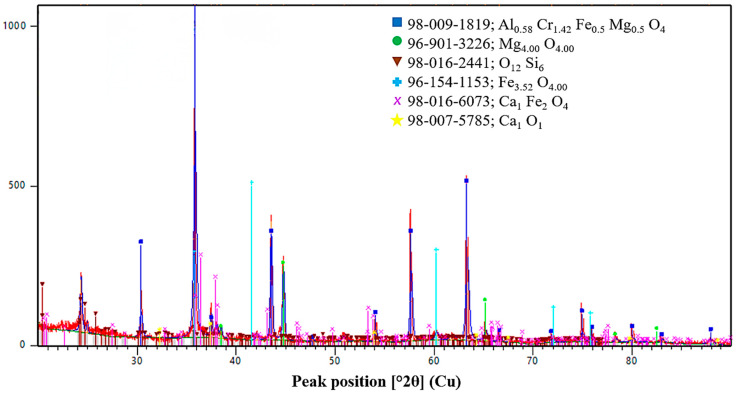
XRD pattern of chromium-bearing material.

**Figure 14:**
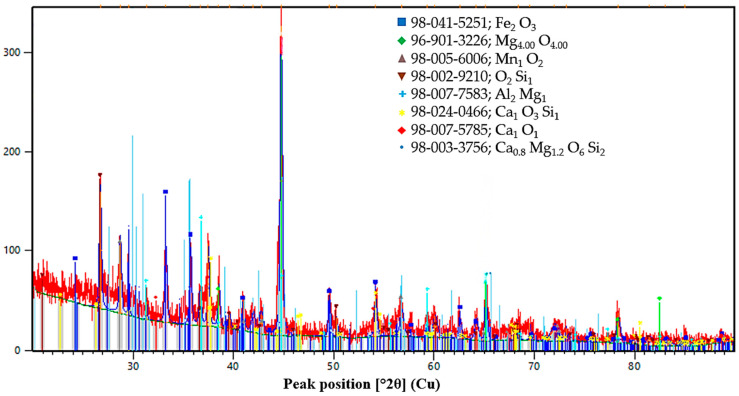
XRD pattern of manganese-bearing material.

**Figure 15:**
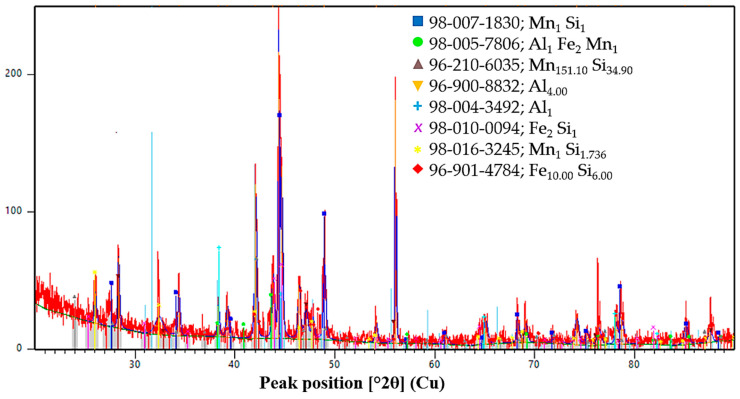
XRD pattern of AlSiMn alloy.

**Figure 16:**
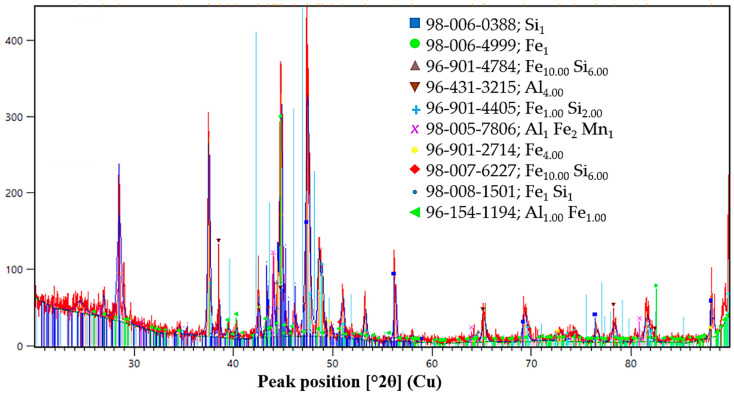
XRD pattern of FeSiAl alloy.

**Figure 17:**
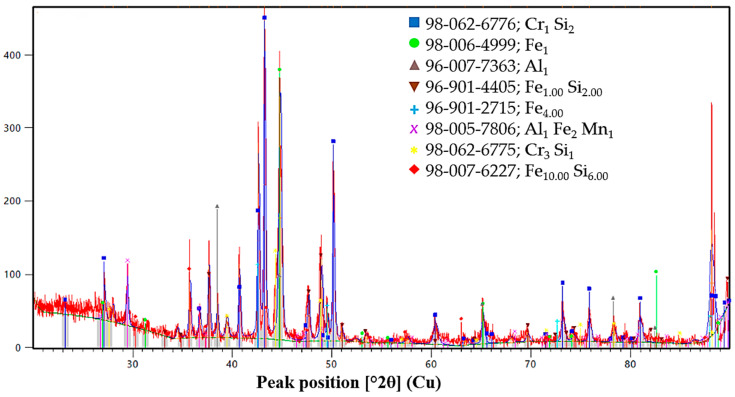
XRD pattern of FeSiCr dust.

The phase state of the initial ores and reductants was characterized by X-ray diffraction analysis, and the corresponding diffraction patterns are presented in Figure 13, Figure 14, Figure 15, Figure 16 and Figure 17. Each material is described in detail below based on the obtained diffractograms.

The phase composition of the chromium ore from the Kempirsay deposit (Figure 13) confirms a complex multiphase oxide system. Chromium is primarily hosted in a high-chromium spinel structure with the composition (Al_0.58_Cr_1.42_Fe_0.5_Mg_0.5_O_4_). High-intensity peaks also correspond to periclase (MgO) and magnetite (Fe_3.52_O_4_), which form the mineralogical matrix of the ore. Regarding the reviewer’s observation on calcium-containing phases, low-intensity reflections for calcium ferrite (CaFe_2_O_4_) and lime (CaO) are clearly identified in the diffractogram. Their presence, despite a total CaO content of <1%, is attributed to the high sensitivity of the XRD equipment and the local crystallization of these components within the silicate gangue. These are classified as trace phases, fully consistent with the chemical analysis data.

The iron–manganese ore from the Kerege-Tas deposit (Figure 14) represents a heterogeneous oxide-silicate system. The primary diffraction peaks correspond to iron oxide (Fe_2_O_3_) and magnesium oxide phases. Manganese is identified in the form of dioxide (MnO_2_), integrated with a silicate matrix. The silicate component includes quartz (SiO_2_) and complex calcium-magnesium silicates such as diopside (Ca_0.8_Mg_1.2_O_6_Si_2_) and wollastonite (CaSiO_3_). The detection of multiple calcium-bearing mineral forms, even at low overall concentrations, is a result of the qualitative XRD analysis capturing the distribution of Ca as a micro-impurity across different silicate phases.

The XRD pattern of the AlSiMn complex reductant (Figure 15) reveals a highly reactive multi-component metallic system characterized by a diverse range of silicide and intermetallic phases. The diffraction data confirms that manganese is primarily present in the form of various silicides, including MnSi, Mn_151.10_Si_34.90_, and MnSi_1.736_, which act as the main carriers of silicon and manganese during the reduction process. The presence of iron–manganese intermetallics, specifically the Al_1_Fe_2_Mn_1_ phase, along with iron silicides such as Fe_2_Si and Fe_10_Si_6_, indicates a high degree of structural complexity that promotes the formation of low-melting-point eutectic phases. Furthermore, the identification of metallic aluminum in Al and Al_4_ forms provides the necessary thermodynamic driving force for the deep reduction of chromium and manganese oxides from the melt.

The XRD pattern of the FeSiAl reductant (Figure 16) reveals a complex phase structure dominated by free silicon (Si) and metallic iron (Fe), which provide the primary reducing potential. Silicon is also present as stable silicides, including Fe_10_Si_6_, FeSi_2_ and FeSi, ensuring controlled reaction kinetics at high temperatures. The identification of metallic Al_4_ and intermetallics like Al_1_Fe_2_Mn_1_ and Al_1_Fe_1_ enhances the system’s chemical activity, facilitating the deep reduction of refractory Cr and Mn oxides while lowering melt viscosity. These phases collectively confirm the high efficiency of FeSiAl for producing multicomponent alloys.

The XRD pattern of the FeSiCr dust (Figure 17) characterizes it as a high-potential secondary reductant with a predominant metallic and silicide phase structure. The primary reducing agents are identified as chromium silicides, specifically Cr_1_Si_2_ and Cr_3_Si_1_, which provide high reactivity during the reduction of transition metal oxides. The diffraction data also confirms a significant presence of metallic iron (Fe_1_, Fe_4.00_) and various iron silicides, such as Fe_1.00_Si_2.00_ and Fe_10.00_Si_6.00_, which facilitate the formation of a stable metallic matrix at high temperatures. Additionally, the identification of the Al_1_Fe_2_Mn_1_ complex intermetallic phase and metallic Al_1_ further enhances the chemical activity of the dust, making it an effective and low-cost component for the synthesis of multicomponent alloys.

### 3.2. Chemical Analysis

The chemical composition of the investigated raw materials and reductants was determined by classical wet chemical analysis in an accredited chemical analysis laboratory of the Institute of Chemistry and Metallurgy. Prior to analysis, the samples were dried, ground, and prepared to the required particle size to ensure homogeneity and representativeness.

The contents of the main components were determined in accordance with standardized laboratory procedures, including sample decomposition in an acidic medium followed by quantitative determination of individual oxides and elements. The applied analytical methods comply with national and international quality standards, and the laboratory is accredited in accordance with ISO/IEC 17025 [33].

The obtained results provide reliable quantitative data on the contents of major oxides in chromium- and manganese-bearing materials, as well as on the elemental composition of silicon- and aluminum-containing reductants. The chemical compositions of the raw materials and reductants are presented in Table 6 and Table 7, respectively.

### 3.3. High-Temperature Experimental Procedure

Experimental studies were carried out using a laboratory-scale induction crucible setup that enables high-temperature treatment of multicomponent oxide–metal systems under controlled conditions. High-temperature experiments were performed for three variants differing in the composition of the initial reaction mixtures. The selection of compositions was based on previously conducted thermodynamic calculations [32], which made it possible to establish rational component ratios for the formation of the Fe–Mn–Cr–Si system.

In the first experimental variant, chromium-bearing ore was used as the sole source of chromium. The initial charge consisted of 100 kg of chromium ore, 30 kg of AlSiMn reductant, and 60 kg of calcium oxide (CaO) as a flux for slag formation.

In the second variant, both chromium-bearing and manganese-bearing ores were employed in equal proportions. The charge included 50 kg of chromium ore, 50 kg of manganese ore, 30 kg of FeSiAl alloy as the reducing agent, and 90 kg of calcium oxide.

In the third variant, manganese-bearing ore was combined with a finely dispersed FeSiCr material used as a silicon-containing reductant. The initial mixture consisted of 100 kg of manganese ore, 50 kg of FeSiCr dust, and 100 kg of calcium oxide.

In all experimental series, calcium oxide served as a flux to form the oxide phase of the required composition.

Induction heating provided intensive and uniform temperature increase in the reaction mixture through the generation of eddy currents in the electrically conductive components of the system. The absence of direct contact between the heating element and the melt minimized contamination and ensured reproducibility of the experimental conditions. The uniform temperature field within the crucible volume enabled accurate registration of thermal effects accompanying the redox reactions.

The experiments were carried out in an alumina crucible with a capacity of 100 g, placed in a graphite holder and a ceramic furnace housing. This configuration ensured effective thermal insulation and geometric stability of the crucible under high-temperature conditions. The operating mode of the setup was maintained at a power of 16 kW and a current of up to 500 A.

**Figure 18:**
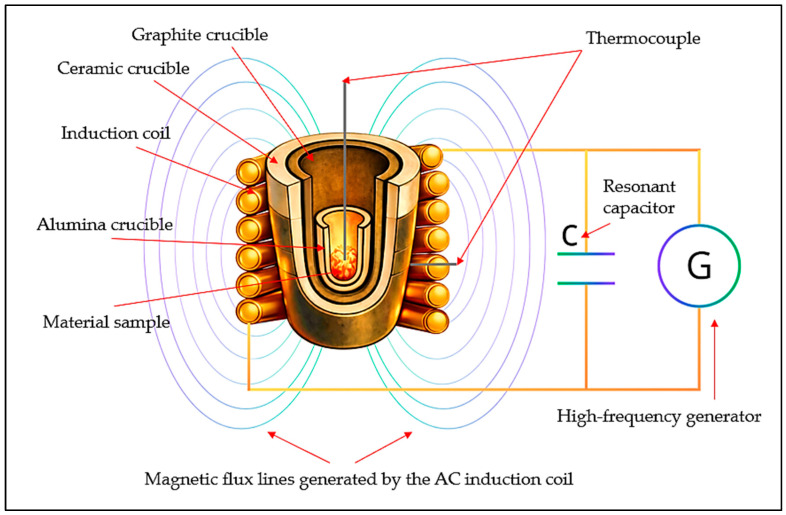
Schematic representation of the laboratory induction crucible setup used for high-temperature experiments and registration of thermal effects.

The temperature regime was monitored using W-Re (5/20) thermocouples (Goodfellow Cambridge Ltd., Huntingdon, UK) positioned near the crucible wall and within the melt zone, allowing the temperature evolution to be recorded during both the heating and holding stages. A water-cooling system for the inductor ensured stable electrical parameters of the setup and prevented overheating of its components. A schematic representation of the experimental setup is provided in Figure 18.

### 3.4. Phase Characterization and Viscosity Measurements

The phase state of the products obtained after high-temperature treatment was investigated by X-ray diffraction. The analysis was performed using a PANalytical X’Pert PRO diffractometer (PANalytical, Almelo, The Netherlands) with Cu Kα radiation (λ = 1.5418 Å) in Bragg–Brentano (θ–2θ) geometry. Diffraction patterns were recorded over a 2θ angular range of 10–90° with a step size of 0.05°. The experimental data were processed using X’Pert HighScore Plus software (PANalytical, Almelo, The Netherlands) with the PDF-2 database (International Centre for Diffraction Data, ICDD).

The microstructural features of the metallic and slag phases, as well as the distribution of major elements, were examined by scanning electron microscopy combined with energy-dispersive X-ray spectroscopy (SEM–EDS). The analyses were carried out using a benchtop scanning electron microscope ZEM20 (ZEPTOOLS, Tongling, China) equipped with an Oxford Instruments EDS detector (Oxford Instruments, Abingdon, UK). The SEM–EDS technique was employed for qualitative analysis of phase morphology and the distribution of chromium, manganese, and associated elements in the investigated samples.

**Figure 19:**
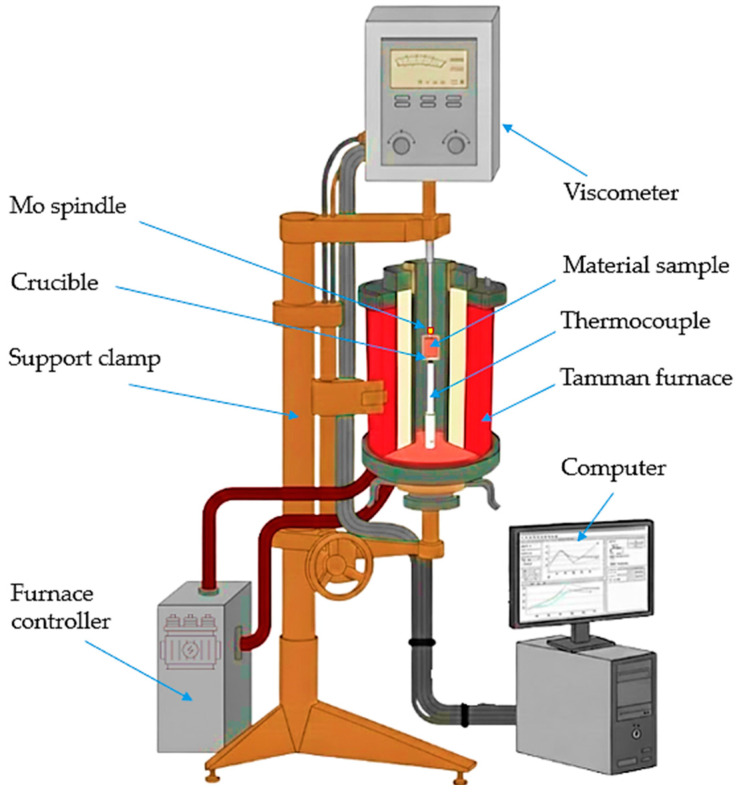
Schematic representation of the high-temperature electrovibrational viscometer setup used for experimental determination of the viscosity of oxide melts.

The viscosity–temperature properties of the slag melts were determined using a high-temperature electrovibrational viscometer in a resistance furnace equipped with a graphite heater (Figure 19). The measurement principle is based on the relationship between the damping of the mechanical vibrations of a submerged probe and the dynamic viscosity of the surrounding melt. Viscosity measurements were performed during the cooling process after ensuring the complete homogenization of the melt. The temperature was continuously monitored using a molybdenum thermocouple positioned in the high-temperature zone in close proximity to the crucible.

For comparison with the experimental data, calculated values of oxide melt viscosity were obtained using the Viscosity module of the FactSage 8.4 software package. The calculations were performed for the corresponding chemical compositions of the slag phase under the temperature conditions applied in the experimental studies.

## 4. Conclusions

In this study, the thermal behavior, phase evolution, and viscosity properties of multicomponent oxide melts in the Cr–Mn system were systematically investigated using an integrated experimental–thermodynamic approach. High-temperature reduction experiments demonstrated that exothermic effects accompanying the reduction of Cr- and Mn-containing oxides manifest as short-lived transient thermal impulses. The intensity of these responses depends on the charge composition and the specific reductant used.

Phase analysis confirmed the formation of multicomponent Fe–Cr–Mn–Si metallic phases, with Chromium and Manganese effectively redistributing into the metallic matrix. Residual concentrations of these oxides in the slag remained at low levels. XRD and SEM–EDS results verified the development of complex metallic structures formed under non-equilibrium conditions, characterized by dendritic microstructures and chemical segregation.

The viscosity behavior of the slags was evaluated through thermodynamic modeling with FactSage 8.4 and experimental high-temperature electrovibrational viscometry using a graphite heater and molybdenum components. It was established that the effective viscosity decreases monotonically with increasing temperature in the investigated range (1400–1650 °C), governed by the thermally induced depolymerization of the silicate–aluminate network. Arrhenius analysis allowed the activation energies of viscous flow (E_a_) and crystallization onset temperatures (T_cr_) to be quantified, confirming that Variant 2, despite its higher initial polymerization, maintains high fluidity at operating temperatures.

Building upon previous research, these results demonstrate that the investigated slag systems exhibit low viscosity, high thermal stability, and a wide homogeneous liquid-phase region. These characteristics ensure favorable conditions for efficient metal–slag separation and stable smelting operations. The findings confirm the technological suitability of the chosen slag regimes for the production of Fe–Cr–Mn alloys, providing a robust physicochemical basis for optimizing high-temperature reduction processes.

## Figures and Tables

**Table 1:** Chemical composition of the metal and slag, wt.%.

No	Metal				Slag						
	Fe	Cr	Mn	Si	CaO	SiO_2_	Al_2_O_3_	MgO	MnO	Cr_2_O_3_	FeO
1	22.92	53.95	19.91	3.20	41.88	21.51	13.59	19.15	0.68	1.97	1.22
2	44.64	23.92	28.21	3.22	53.60	26.47	9.10	7.09	1.42	1.09	1.24
3	35.56	20.34	39.51	4.59	59.35	35.51	1.81	0.10	0.54	1.69	1.01

**Table 2:** Results of EDS elemental analysis (V1).

Spectrum No	Content, wt., %			
	Fe	Cr	Mn	Si
1	19.21	69.63	6.88	3.98
2	8.49	63.46	21.32	6.73
3	29.11	44.39	22.51	3.99

**Table 3:** Results of EDS elemental analysis (V2).

Spectrum No	Content, wt., %			
	Fe	Cr	Mn	Si
1	48.36	23.38	23.13	5.13
2	38.93	16.74	38.22	6.11
3	36.37	52.16	8.15	3.32

**Table 4:** Results of EDS elemental analysis (V3).

Spectrum No	Content, wt., %			
	Fe	Cr	Mn	Si
1	39.98	16.72	39.29	6.01
2	24.39	12.13	61.32	2.16
3	41.77	26.32	27.03	4.88

**Table 5:** Arrhenius parameters, activation energy of viscous flow, and crystallization onset temperatures for the investigated slag compositions.

No	B	T_cr._, °C	Arrhenius Equation	E_a_, kJ/mol
1	1.74	1415	ln_η_ = −12.05 + 22,831/T	189.814
2	1.69	1380	ln_η_ = −10.68 + 21,148/T	175.822
3	1.66	1435	ln_η_ = −10.21 + 19,455/T	161.748

**Table 6:** Chemical composition of the initial charge materials.

Materials	Content, wt.%								
	Cr_2_O_3_	Mn_2_O_3_	Fe_2_O_3_	SiO_2_	Al_2_O_3_	MgO	CaO	P_2_O_5_	S
Cr ore	39.86	-	11.84	10.93	8.85	27.85	0.64	0.01	0.01
iron-Mn ore	-	38.29	39.87	16.74	2.57	0.15	2.34	0.03	0.01

**Table 7:** Chemical composition of the reductants.

Materials	Content, wt.%						
	Cr	Mn	Fe	Si	Al	S	P
AlSiMn	-	29.04	13.97	48.39	8.55	0.020	0.021
FeSiAl	-	-	40.52	48.62	10.82	0.010	0.024
dust FeSiCr	24.10	-	42.10	32.22	1.51	0.049	0.018

## Data Availability

The data supporting the findings of this study are available from the corresponding author upon reasonable request.
